# Platelets and Their Role in the Pathogenesis of Cardiovascular Events in Patients With Community-Acquired Pneumonia

**DOI:** 10.3389/fimmu.2020.577303

**Published:** 2020-09-17

**Authors:** Charles Feldman, Ronald Anderson

**Affiliations:** ^1^Department of Internal Medicine, Faculty of Health Sciences, University of the Witwatersrand, Johannesburg, South Africa; ^2^Department of Immunology, Faculty of Health Sciences, Institute of Cellular and Molecular Medicine, University of Pretoria, Pretoria, South Africa

**Keywords:** anti-platelet agents, community-acquired pneumonia, pneumococcus, platelets, pneumolysin, thrombocytopenia, high mobility group box 1 protein, cardiovascular events

## Abstract

Community-acquired pneumonia (CAP) remains an important cause of morbidity and mortality throughout the world with much recent and ongoing research focused on the occurrence of cardiovascular events (CVEs) during the infection, which are associated with adverse short-term and long-term survival. Much of the research directed at unraveling the pathogenesis of these events has been undertaken in the settings of experimental and clinical CAP caused by the dangerous, bacterial respiratory pathogen, *Streptococcus pneumoniae* (pneumococcus), which remains the most common bacterial cause of CAP. Studies of this type have revealed that although platelets play an important role in host defense against infection, there is also increasing recognition that hyperactivation of these cells contributes to a pro-inflammatory, prothrombotic systemic milieu that contributes to the etiology of CVEs. In the case of the pneumococcus, platelet-driven myocardial damage and dysfunction is exacerbated by the direct cardiotoxic actions of pneumolysin, a major pore-forming toxin of this pathogen, which also acts as potent activator of platelets. This review is focused on the role of platelets in host defense against infection, including pneumococcal infection in particular, and reviews the current literature describing the potential mechanisms by which platelet activation contributes to cardiovascular complications in CAP. This is preceded by an evaluation of the burden of pneumococcal infection in CAP, the clinical features and putative pathogenic mechanisms of the CVE, and concludes with an evaluation of the potential utility of the anti-platelet activity of macrolides and various adjunctive therapies.

## Introduction

A number of recent reviews, published by authors from different regions of the world, including North America, Latin America, Europe and Africa, attests to the fact that, globally, community-acquired pneumonia (CAP) remains a common cause of hospitalization, morbidity, mortality, and health-care costs (1–5). There are, however, notable variations in the burden and epidemiology of CAP in different regions of the world. These result from a number of factors, such as differences in population aging, increasing incidence of comorbidities, prevalence of smoking, antibiotic resistance, the introduction of pneumococcal conjugate vaccination (PCV) in children, and the use of pneumococcal and influenza vaccination in adults. Moreover, regional differences in the reported microbial etiology of CAP exist that are related, at least in part, to variations in the diagnostic methods used to identify the causative pathogens (1–5). Irrespective of these regional differences in prevalence and reported etiology, it is abundantly evident from most publications on this important public health issue that the clinical and economic burden of CAP “…is staggering, far-reaching, and expected to increase in the future…” (1).

The main aim of the current review, which fits with the theme of this special issue of “Frontiers in Immunology,” is to describe the emerging role of platelets in the pathogenesis of cardiovascular (CV) complications in patients with severe CAP, a major cause of morbidity and mortality in this condition. This encompasses not only CV complications in the setting of acute disease, particularly that caused by *Streptococcus pneumoniae* (the pneumococcus), but also the long-term sequelae of severe CAP and its apparent associations with persistent antigenemia and inflammation.

## Burden of CAP

The Global Burden of Disease (GBD) Study, which evaluated the impact of lower respiratory tract infections (LRTIs; pneumonia and bronchiolitis) in 195 countries (6), documented in 2015 that these infections accounted for some 2.74 million cases (95% uncertainty interval [UI] of 2.5 million to 2.86 million). While the burden of LRTIs decreased dramatically between 2005 and 2015 in children younger than 5 years, the burden in adults >70 years has however, increased in many regions. With regard to etiology, some authors have noted that the prevalence of pneumococcal CAP appears to be decreasing in North America, but not in other regions of the world, such as Europe, and have attributed this to differences in vaccination rates and smoking habits (7). In the 2015 GBD study, the pneumococcus was the most common cause of LRTIs on a global scale, causing more deaths than all of the other studied respiratory pathogens combined, including *Haemophilus influenzae*, influenza virus and rhinovirus (6). Additional regional and national studies, such as those from the Eastern Mediterranean region (8) and Brazil (2, 9), supported by investigations from Europe (10), confirm the predominance of the pneumococcus as a cause of CAP.

Importantly, mortality rates of patients with CAP remain very high (11). Indeed, when reviewing the literature, it becomes apparent that mortality in CAP may occur early, described as occurring within the first 72 h to 7 days after hospital admission, or over the short term, commonly measured as the first 28–30 days after diagnosis or hospital admission (11). Importantly, even over the long term, as measured in months or years after hospital discharge, the mortality of CAP patients remains very high, being greater than that of patients admitted to the medical ward for conditions other than CAP, even when adjusted for age and comorbidity (11). While underlying co-morbidities may be important risk factors, there is increasing awareness that the occurrence of cardiac complications, even those occurring while the patient is in hospital, may impact not only on immediate and short-term mortality, but also on long-term mortality in patients who have survived an episode of CAP. In this context, a number of studies have investigated the utility of various biomarkers in predicting both the early and long-term mortality of CAP. Although elevated levels of several biomarkers of cardiovascular disease (CVD) or coagulation, measured either in hospital, or on hospital discharge of patients apparently recovered from acute CAP, were significantly associated with poorer outcomes, the significance of these findings is not entirely understood (11, 12). Do these biomarkers simply indicate the presence of underlying cardiac comorbidity, or are they non-specific markers of inflammation in CAP, or could they be indicators of acute cardiac injury due to CAP (11, 13)?

## Cardiac Complications in All-Cause CAP

The occurrence of CV complications as a consequence of both acute bacterial and viral infections, has been known for some time (14, 15); however, it is now well-recognized that cases admitted with infections such as pneumonia and sepsis have an increased incidence of CV conditions such as acute myocardial infarction (AMI) or stroke, not only during hospital admission, but also occurring after hospital discharge. In one study comparing hospitalized patients with and without sepsis, the former patients had an increased incidence of AMI or stroke [(adjusted odds ratio 1.72; 95% confidence interval (CI) 1.60–1.85)], which occurred within 70 days of hospital discharge and was associated with an increased 180 day and 1 year mortality (15). Following adjustment for age, sex and comorbidity, these CV events (CVEs) were found to be independent risk factors for 180 day mortality.

Some studies have shown that both *S. pneumoniae* and influenza virus were particularly important in triggering a CVE (16, 17). However, one recently published abstract suggested that the frequency of major CVEs, referred to as MACE, and encompassing all-cause mortality, non-fatal AMI, stroke and heart failure, occurring within the first 90 days was 60% higher in CAP patients diagnosed as having a bacterial infection compared to those with viral pneumonia (17). The occurrence of these CAP-related complications is not surprising, since severe sepsis occurs early in the course of this infection in more than 30% of cases, and while it may involve multiple organ systems, it commonly involves the heart (18). In fact, some reviews have labeled pneumonia as a CVD (19). Clearly, there is no doubt that the occurrence of CVEs, is common, and after adjusting for confounders, is a significant risk factor for in-hospital mortality, as well as increased short-term mortality (20, 21).

Besides an increase in mortality, patients with CAP who develop CV dysfunction in hospital also have a significantly higher requirement for mechanical ventilation, need for inotropes and vasopressors, a higher rate of development of acute renal failure, and a more prolonged hospital stay, compared to CAP patients without a cardiac event (22). While the risk of AMI is greatest early in the course of infection and is related to the severity of infection, importantly it persists beyond the short-term infection period, exceeding the baseline risk even up to 10 years after infection (14).

Clearly, the major long-term negative outcome consequences in patients who have survived an episode of CAP are the occurrence of CVEs and mortality, which, as indicated above, are interrelated. Accordingly, prediction of these events early in the course of infection would represent a major advance that could enable early intervention. To this end, various investigators have assessed the utility of biomarkers, both systemic and cardiac, and risk stratification tools, such as severity of illness scores, as well as a combination of these as strategies to optimize outcome prediction in CAP patients (12, 23, 24). Given the absence of an ideal, single prognostic biomarker, current evidence suggests that measurement of a combination of biomarkers in CAP patients may offer the best predictive strategy. This involves combining measurement of the systemic inflammatory biomarkers, procalcitonin (PCT) and C-reactive protein (CRP), as indices of disease severity and short-term outcome assessment, with biomarkers of CV risk such as N-terminal pro-brain natriuretic peptide (proBNP), mid-regional pro-adrenomedullin (proADM), endothelin and troponins, together with severity of illness scores, such as the CURB-65 score (12, 23–25). In this setting, it is noteworthy that the CHA2DS2-VASc score has been found to be a good predictor of new-onset atrial fibrillation in patients hospitalized for CAP (25).

## Cardiac Complications in Pneumococcal CAP

Musher et al. were the first to describe the occurrence of acute cardiac events in patients with pneumococcal CAP (26). They described 170 patients hospitalized with pneumococcal CAP, of whom 33 (19.4%) had one or more of the major cardiac events that have been described in patients with CAP; 12 patients had AMI [of whom two also had arrhythmia and five had new-onset or worsening congestive heart failure (CHF)]; eight had new-onset atrial fibrillation or ventricular tachycardia (of whom six also had new-onset CHF); and 13 had newly-diagnosed or worsening CHF on its own. While these are the classical cardiac changes described in patients with pneumococcal CAP, Chiong et al. have, more recently, described a teenager, with bacteremic pneumococcal pneumonia, who presented with features suggestive of an ST-elevation acute MI (STEMI) and cardiogenic shock that was due to myocarditis and rhabdomyolysis (27).

Several other recent studies have shown that (CVEs) occur relatively frequently in patients with pneumococcal CAP, which is the cause of CAP that is best studied with regard to pathogenic mechanisms (13, 28, 29). This contention is underscored by the findings of a recent secondary analysis of data from an international, multicenter, observational, cohort study involving 18 hospitals in 7 countries, encompassing 2,088 patients, 921 (44%) of whom had bacteremic pneumococcal pneumonia and 1,167 (55.9%) non-bacteremic pneumococcal pneumonia (30). Cardiovascular events were noted in 275 (13%) patients with a significantly higher incidence in bacteremic vs. non-bacteremic cases (15% vs. 12%; *p* = 0.02). There were 316 CVEs in total, of which 25 (7.9%) were AMI, 150 (47.5%) new and 51 (16.1%) worsening arrhythmia, and 51 (16%) new and 39 (12.4%) worsening heart failure. The rate of new-onset cardiac arrhythmias was also significantly higher in the bacteremic group.

With respect to the pathogenesis of CVEs in severe pneumococcal CAP, a number of very recent studies, mostly involving different animal models of experimental pneumococcal infection, have documented that the pneumococcus is able to translocate across the vascular endothelium and invade the myocardium (31–33). Once in the heart, the microorganism kills myocytes, as well as resident and infiltrating macrophages, via activation of necroptosis, causing significant damage to the myocardium that results in tissue remodeling (31–33). In their study, Shenoy et al. using a murine model of invasive pneumococcal disease, demonstrated that cardiac damage only occurred in the setting of infection with those pneumococcal serotypes that caused high-grade bacteremia (31). Cardiac damage was assessed according to elevated serum cardiac troponin-I levels, as well as cardiac histology that varied according to pneumococcal serotype (31). These findings in the experimental setting confirm those of the clinical study reported by Borsa et al. in patients with bacteremic and non-bacteremic pneumococcal pneumonia (30).

Cardiac damage in the setting of invasive experimental disease appears to be caused by pro-inflammatory mechanisms triggered by pneumococcal cell wall components, as well as by pneumolysin, the thiol-activated, pore-forming toxin of the pathogen (13, 28, 31, 34). Pneumolysin-mediated cardiac damage may result from the direct cardiotoxic/immunosuppressive activities of the toxin, as well as from its indirect pro-inflammatory/prothrombotic activities (28). In addition, work from our laboratory, and that reported by other investigators, has suggested that platelet activation and platelet-driven neutrophil activation may also contribute to the pathogenesis of these acute cardiac events (13, 35).

The following section of this review encompasses the role of platelets, both protective and harmful, in anti-bacterial host defense in the setting of CAP, most prominently in relation to the pneumococcus.

## Platelets and Host Defense

Platelets originate during thrombopoiesis in the bone marrow, a process regulated by the hormone, thrombopoietin, produced in the liver and kidney (36). During thrombopoiesis, small, anuclear pro-platelets originate from marrow-derived megakaryocytes. Adult humans produce platelets at a steady state of 10^11^ cells/daily and maintain circulating levels of these cells at between 150 and 450 × 10^9^ platelets per liter (L) of blood, although the tempo of production may increase in times of heightened demand by 20-fold or even more (36). In the circulation, platelets have a lifespan of 7–10 days that is determined by the balance between the cellular levels of the anti-apoptotic and pro-apopototic factors, Bcl-x(L) (B cell lymphoma-extra large) and Bak [protein encoded by the BAK1 gene (BCL2 antagonist/killer1)], respectively (37). Although circulating platelet counts decline with age, somewhat paradoxically, this decrease in the numbers of these cells is associated with acquisition of a pro-inflammatory phenotype (38). The age-related transition of platelets to a more reactive state is associated with increased generation of reactive oxygen species (ROS), mitochondrial dysfunction and activation of the mammalian target of rapamycin (mTOR) pathway (38). In addition, the serine esterase, granzyme A, has recently been detected in non-α-granule, sub-membranous areas of human platelets, attaining levels that are up to 9-fold higher in platelets from older as opposed to younger adults (39). Platelet-derived granzyme A also appears to contribute to the pro-inflammatory phenotype of aging platelets via activation of Toll-like receptor (TLR) 4- and caspase-1-dependent mechanisms that potentiate the synthesis of the monocyte-derived chemokines, interleukin (IL)-8 (CXCL8) and monocyte chemoattractant protein-1 (MCP-1, CCL2) (39).

These age-related alterations in the numbers and reactivity of circulating platelets are likely to reflect the chronic, low-grade, systemic inflammation that is associated with age-related co-morbidities (39). This type of sub-clinical, intravascular instability may predispose elderly adult humans for development of severe organ dysfunction, or even failure, during episodes of serious microbial or viral infection.

Putative mechanisms underpinning these threats, specifically in the context of serious CVEs in patients with invasive CAP, are covered in a later section of this review.

## Platelets and Infection

Although the primary function of the circulating platelet is to maintain homeostasis through continuous monitoring of vascular endothelial integrity (36), platelets also play a key role in the systemic recognition and control of invasive microbial and viral pathogens. As highlighted in a series of recent reviews, platelets trap, sequester and, in some cases, eliminate invasive pathogens (40–47). Importantly, platelets also enlist assistance from more powerful cellular components of host defense. In this context, by trapping, weakening and immobilizing invasive pathogens, facilitating their access to neutrophils and monocytes, platelets coordinate and amplify the protective activities of the cellular and humoral elements of both the innate and adaptive immune systems (40–47).

Mechanisms utilized by platelets to counter the threat posed by pathogens that breach local host defenses at sites of primary infection have recently been covered in detail elsewhere (43–47). Accordingly, only direct mechanisms of antimicrobial activity mediated by platelet-derived anti-infective peptides/polypeptides, as well as indirect mechanisms involving high mobility group box 1 (HMGB1) protein, have been included here. Thereafter, the remaining sections of this part of the review focus on mechanisms and adverse consequences of systemic over-reactivity of platelets in the setting of CAP caused by highly invasive bacterial pathogens, particularly the pneumococcus.

### Mechanisms Utilized by Platelets to Trap Pathogens During Invasive CAP

Despite their small size (2–3 μm diameter), platelets are endowed with abundant cytoplasmic granules containing an array of pre-formed mediators with a range of biological activities. Alpha-granules predominate in the platelet cytosol, numbering 50–80/cell, while dense granules that number 3–5/cell and small numbers of T granules (contain TLR9 and protein disulfide isomerase) and lysozomes (contain acid hydrolases) are also present (48). Platelet activation occurs most prominently following exposure to thrombin, adenosine 5′-diphosphate (ADP), thromboxane A_2_, fibronectin and collagen; these platelet activators interact with proteinase-activated receptors 1 and 4, P2Y1 and P2Y12 purinergic receptors, the TP2 prostanoid receptor, the GPIIb/IIIa integrin and the α_2_β1 integrin/GPVI, respectively. Following activation, platelets undergo degranulation that results in the release of various cytokines, chemokines, pro-coagulants, pro-angiogenic mediators and growth factors that either act extracellularly, or replenish membrane receptors (45, 47). These various mediators initiate key platelet pro-thrombotic/pro-inflammatory activities, including intercellular adhesion (endothelial cells, neutrophils and monocytes), homotypic aggregation, autocrine activation, coagulation, migration, and, in the context of this review, pathogen capture and antimicrobial activity.

#### Platelet Receptors Involved in Trapping of CAP Pathogens

Platelets possess a range of mechanisms that enable trapping of invasive CAP pathogens, as well as other types of bacterial pathogens (40, 47, 49). Some are expressed constitutively while others are triggered during platelet activation. These mechanisms include:

- expression of the FcγIIA receptor, which interacts with pathogen-bound antibodies of the IgG_2_ subclass (49), a mechanism that may be of particular relevance in the context of capsular polysaccharide-expressing pathogens such as the pneumococcus and *Haemophilus influenzae*;
- expression of complement receptors, specifically cC1qR and gC1qR that interact with pathogen-bound, activated complement component C1q, as well as receptors for C3a and C5a, which may be potentiated by discharge of platelet α-granule-derived C3 (50);
- binding of *Staphylococcus aureus* and various streptococcal species to platelet glycoprotein 1b (GP1b) via surface protein adhesins, or, in the case of *S. aureus*, by an additional, albeit indirect, mechanism involving von Willebrand factor (40);
- expression of a range of Toll-like receptors (TLRs 1, 2, 3, 4, 6, 7, and 9) that have the potential to trap invasive bacterial and viral pathogens (51); TLR2, via interactions with lipoteichoic acids and peptidoglycan, as well as TLR4 interactions with bacterial lipopolysaccharide, have the potential to entrap Gram-positive and Gram-negative bacteria, respectively. In the case of viral pathogens, platelets capture influenza virus particles via a TLR7-dependent mechanism (52). Although this mechanism may be protective, it also poses the risk of vascular occlusion due to release of C3 from platelets and excessive neutrophil activation (52).

With respect to the pneumococcus, “selective” interaction of this pathogen with platelets may be mediated by several putative mechanisms. These involve interaction of: (i) cell-wall phosphorylcholine with the platelet-activating factor receptor (PAFR) (53); (ii) the pneumococcal adhesins, PavB and PavC, with the GPIIb/IIIa(α2b/β3a)/fibrin/thrombospondin-1 complex (54); (iii) the phage-encoded pneumococcal adhesins, pblA and pblB, with platelet membrane gangliosides (55); and (iv) activation of TLR4 by pneumolysin, as well as by toxin devoid of cytolytic activity (56). Moreover, it is noteworthy that platelets are activated by hydrogen peroxide (57, 58), which is produced in large amounts by the pneumococcus, representing a putative, albeit unproven, additional mechanism of activation of these cells by the pathogen.

These various mechanisms of pathogen/platelet interactions, several of which result in platelet activation, expose the pathogens to the anti-infective mechanisms of the platelet, mostly antimicrobial peptides/polypeptides located predominantly, but not exclusively, in α-granules.

### Granule-Derived Mechanisms Utilized by Platelets to Neutralize Microbial Pathogens

Platelet granule-derived, anti-infective peptides/polypeptides are mostly cationic amphiphiles with broad-spectrum activity against microbial and viral pathogens. They include α- and β-defensins, kinocidins, thrombocidins, and thymosin β-4.

#### Platelet Defensins

Human platelets and megakaryocytes have recently been reported to express defensin α-1 (DEFA1), also known as human neutrophil peptide-1 (HNP1), which co-localizes with platelet α-granules and has also been found in the cytoplasm of a megakaryoblastic leukemia cell line (59). On exposure to activators such as thrombin, ADP or lipopolysaccharide, DEFA1 relocates to the platelet outer surface where it exhibits anti-bacterial activity against the Gram-negative bacterial pathogen, *Escherichia coli*, confirming its involvement in platelet-mediated antimicrobial activity (59).

Platelets apparently also contain two human β-defensins (HBDs), namely HBD1 and HBD3, usually found in epithelial cells (60). In the case of HBD1, mRNA encoding this antimicrobial peptide, as well as the intact protein, are located in platelet extra-granular cytoplasmic compartments (60). In keeping with a non-granule intracellular location, extracellular discharge of HBD1 did not occur following exposure of platelets to receptor-mediated mobilization of cytoplasmic granules (60). Exposure of platelets to the pore-forming α-toxin of *S. aureus*, however, did induce release of HBD1, which was associated with inhibition of the growth of various clinical strains of this pathogen (60). In addition, HBD1 induced the formation of neutrophil extracellular traps (NETs) as an auxiliary mechanism of antimicrobial activity (60). Interestingly, proteolysis of the reduced, active form of HBD1 resulted in the formation of an octapeptide with significantly increased antimicrobial potency and a broader spectrum of activity, encompassing antibiotic resistant organisms including *E. coli, Pseudomonas aeruginosa*, and *Candida albicans* (61).

In the case of HBD3, the evidence in support of an intracellular localization of this antimicrobial peptide is somewhat less convincing, as it is based on the presence of this anti-infective agent in human platelet concentrates, as opposed to isolated cells, with no evidence provided in support of intracellular detection and location (62). Although interesting, further exploration is necessary to establish or refute the role of HBD3 in platelet anti-infective activity.

#### Kinocidins

These are primarily chemokines, specifically CXCL4 [also known as platelet factor 4 (PF4)], CXCL7 [also known as platelet basic protein (PBP)] and CCL5 (also known as Regulated on Activation, Normal T Cell Expressed and Secreted (RANTES)] that possess secondary antimicrobial activities, with CXCL4 appearing to be the most potent of these (63–66). Platelet kinocidins are vulnerable to proteolytic cleavage by thrombin, resulting in the generation of smaller antimicrobial peptides known as thrombocidins that are C-terminal deletion products of CXC chemokines (64, 65). In this context, CXCL7 undergoes proteolytic modification to generate two antimicrobial peptides known as thrombocidin-1 (TC-1) and thrombocidin-2 (TC-2) (67). Kinocidins and thrombicidins are present in platelet α-granules and, like defensins, they are amphipathic, cationic antimicrobial agents that promote membrane disruption in target pathogens (68).

#### Thymosin Beta-4

Platelet α-granules also contain high concentrations of the ubiquitous 43-amino acid reparative, actin-binding polypeptide, thymosin β-4 (TB4), which also possesses antimicrobial properties (65, 69, 70).

### High Mobility Group Box 1 (HMGB1) Protein and Platelets

HMGB1 serves a critical role in nucleated cells by maintaining genomic stability and structural integrity (71). Somewhat surprisingly, however, anucleate platelets represent a major source of HMGB1, underscoring the existence of roles for this protein beyond maintaining nuclear stability (72, 73). In this context, megakaryocytes synthesize and deliver both HMGB1 and its encoding mRNA to platelets, where both have a cytosolic location (73). Following receptor-mediated activation of platelets by recognized agonists, mobilization of α-granules results in upregulation of expression the adhesion molecule, CD62P (P-selectin). This event, in turn, promotes the formation of platelet/leucocyte (neutrophils and monocytes) heterotypic aggregates, as well as binding of platelets and platelet aggregates to vascular endothelial cells. Cellular adhesion is achieved via expression of the counter receptor for CD62P, namely P-selectin glycoprotein ligand 1 (PSGL-1). These platelet-driven cellular interactions result in the translocation of HMGB1 from the cytosol of these cells to the plasma membrane where it interacts with several types of pro-inflammatory receptors expressed on adherent neutrophils, monocytes and endothelial cells, as well as with platelets *per se*, causing secondary autocrine activation of these cells. These receptors are TLR4 and RAGE (receptor for advanced glycation end products) (73, 74). In addition to interacting with these receptors, HMGB1 also functions as a ligand for TREM-1 (triggering receptor expressed on myeloid cells 1), a receptor expressed on neutrophils and monocytes that amplifies TLR4- and RAGE-mediated intracellular signaling (75).

Importantly, HMGB1 is a composite protein that exists as different isoforms determined by the redox status of the three cysteine residues, namely C23, C45, and C106. The fully oxidized isoform is seemingly biologically inactive. However, the remaining two isoforms possess distinct pro-inflammatory activities. These include firstly, an isoform of HMGB1 that results from selective oxidation of the proximal cysteines, C23 and C45, resulting in formation of an intramolecular disulfide bond, while C106 remains in the reduced state (76). These redox modifications enable this variant of HMGB1 to function as a ligand for TLR4 (77). However, the predominant isoform of HMGB1with respect to functioning as an effective ligand for RAGE and TREM-1 has not been identified (78, 79).

Secondly, an isoform in which all three cysteine residues are unmodified, this being the fully reduced form (76, 80). This isoform of HMGB1 forms a heterocomplex with the chemokine, CXCL12 (stromal-derived factor, SDF-1) (81), that is present in platelet α-granules (82). Interaction of CXCL12 with the fully reduced isoform of HMGB1 augments the affinity of the chemokine for its receptor, CXCR4, thereby amplifying recruitment of immune and inflammatory cells (81).

In the case of neutrophils, engagement of platelet-derived HMGB1 with TLR4 or RAGE has been reported to promote the formation of NETs (83, 84), which, if well controlled, may also contribute to intravascular host defenses.

#### Platelets, HMGB1, Neutrophils, and Reactive Oxygen Species

Like neutrophils, platelets possess the NOX2 isoform of NADPH oxidase. However, the levels of superoxide and H_2_O_2_ produced by platelets following activation with thrombin are apparently 1,500- and 4,000-fold lower than those generated by activated neutrophils (85). Nevertheless, as described in several earlier studies, this low level of production of ROS does appear to be important in augmenting activation of the fibrinogen/fibronectin-binding integrin, GPIIb/IIIa, following activation of platelets with thrombin (86, 87), a contention that has, however, been challenged in a more recent study (88). Accordingly, ROS derived from platelet-activated neutrophils may predominate with respect to augmentation of platelet activation. In this context, platelet-derived HMGB1 activates neutrophil NOX2, both *in vitro* and *in vivo*, by a TLR4-dependent mechanism (89, 90). These neutrophil-targeted, pro-oxidative activities of HMGB1 may further amplify neutrophil/platelet heterotypic interactions as a result of ROS-mediated, Ca^2+^-dependent activation of the neutrophil integrin, αMβ2 (Mac-1; CR3) (91), via interaction with its recently identified alternate ligand, PF4 (CXCL4), expressed on the platelet membrane (92).

Table 1 summarizes the mechanisms of direct and indirect platelet-mediated anti-infective activity involving antimicrobial peptides/polypeptides and HMGB1, respectively.

**Table 1:** Platelet-derived mediators of antimicrobial activity.

**Mediator**	**Location**	**Mechanism of action**	**References**
Defensin α-1 (DEFA1)	α-granules	Direct, membrane-targeted, broad-spectrum activity	(59)
Defensin β-1 (HBD1)	Extragranular, cytoplasmic location	Direct, membrane-targeted broad-spectrum activity, potentiated by proteolytic modification resulting in formation of an octapeptide; also potentiates antimicrobial activity indirectly by induction of NETosis	(60, 61)
Defensin β-3 (HBD3)	Unknown	Role, if any, in platelet antimicrobial activity awaits clarification	(62)
Kinocidins (CXCL4, CXCL7, CCL5)	α-granules	Direct, membrane-targeted broad-spectrum activity	(63–68)
Thrombocidins such as TC-1 and TC-2 generated by thrombin-mediated clearance of CXCL7	α-granules extracellular	Direct, membrane-targeted broad-spectrum activity	(67, 68)
Thymosin β-4	α-granules	Direct, membrane-targeted broad-spectrum activity	(65, 69, 70)
HMGB1	cytoplasmic	Indirect mechanisms of antimicrobial action including induction of NETosis and activation of neutrophil NOX2	(83, 84, 89, 90)

#### Platelets, HMGB1, and Protection Against Bacterial Infection in the Experimental Setting

Zhou et al. using a murine model of intra-abdominal sepsis induced by cecal ligation and puncture have recently described the involvement of platelet-derived HMGB1 in countering the threat posed by invasive bacterial infection (90). To explore, more definitively, the role of platelet HMGB1 in the pathogenesis of experimental sepsis, specifically clearance of bacteria from the peritoneum and circulation, these authors generated gene knockout mice with selective deletion of the genes encoding either HMGB1 individually, or the combination of HMGB1/PF4, in megakaryocytes (90). Using their model of polymicrobial sepsis, the authors observed that mice with HMGB1/PF4-depleted platelets demonstrated significantly greater mortality than wild-type mice that was associated with increased bacterial loads in the peritoneum and blood, as well as an intense systemic inflammatory response (90). Mice harboring platelets selectively depleted of HMGB1 demonstrated decreased levels of the platelet-derived chemokines, RANTES and PF4 in the peritoneal cavity, as well as decreased platelet/neutrophil interaction in the airways. With respect to *in vitro* studies, exposure of neutrophils to platelets genetically depleted of HMGB1 resulted in significant impairment of formation of neutrophil/platelet aggregates and production of ROS by neutrophils (90).

The authors concluded that these observations revealed the previously unrecognized involvement of platelet-derived HMGB1 in the regulation of neutrophil recruitment and activation by modulating platelet activation during sepsis (90), presumably augmented via the participation of PF4.

### Clinical Studies on the Role of Platelets in Protection Against Invasive Bacterial Infections

Claushuis et al. have described clinical studies supporting the involvement of platelets in protecting against invasive bacterial infection. In 2016, these authors reported a retrospective analysis of a study undertaken between 2011 and 2013 that was focused on the relationship between circulating platelet counts and mortality in critically ill sepsis patients (*n* = 929) (93). Thrombocytopenia, particularly very low circulating platelet counts of <50 × 10^9^/L blood, detected in patients with severe sepsis on admission to a hospital intensive care unit, was associated with intense systemic inflammation and significantly increased mortality relative to those patients with intermediate and normal platelet counts (93). More recently, the same authors reported a similar type of retrospective analysis undertaken in Thailand between 2002 and 2006, in which they investigated relationships between circulating platelet counts and mortality in a cohort of patients (*n* = 1,160) hospitalized with culture-proven melioidosis (94). This infective condition presents as a severe pneumosepsis caused by the Gram-negative bacterial pathogen, *Burkholderia pseudomallei*, which is common in Southeast Asia and has a mortality rate of up to 40% (94). The authors again reported that the presence of thrombocytopenia measured at the time of hospital admission was predictive of mortality (94).

To explore mechanisms underpinning the involvement of platelets in disease pathogenesis, the same authors developed a murine model of experimental melioidosis (94). This model revealed the following: i) experimental infection with *B. pseudomallei* resulted in decreased circulating platelet counts and increased mortality that was enhanced by administration of an anti-platelet antibody targeted against the von Willebrand factor-binding glycoprotein, GPIbα; and ii) early lung bleeding that was associated with altered vascular integrity (94). This latter observation is consistent with the role of platelets in maintaining vascular endothelial integrity, especially in preventing vascular leakage during neutrophil extravasation (95).

Kirby et al. confirmed the findings of the clinical component of the aforementioned study (96). These Australian investigators also conducted a retrospective analysis covering the period 1999–2017, encompassing patients (*n* = 758) with proven melioidosis. These authors reported that patients who experienced bacteremia or septic shock had significantly lower platelet counts (227 × 10^9^/L and 217 × 10^9^/L, respectively) than those who did not (corresponding values of 323 × 10^9^/L and 266 × 10^9^/L; *p* < 0,001 and *p* = 0,002, respectively). Although found to be an independent predictor of mortality, the authors concluded that the platelet count is not a useful biomarker to predict severity of melioidosis as most patients with severe disease still had counts that were within the normal range (96).

### Pathogen-Mediated Chronic and Hyperacute Activation of Platelets

The evidence presented in the preceding sections of this review is clearly supportive of a protective role for tightly controlled activation of platelets in anti-infective host defense in the setting of maintenance of vascular integrity. This contention must, however, be balanced against evidence to the contrary, which has implicated platelets in the pathogenesis of organ dysfunction in both chronic and acute inflammatory disorders of infective origin, including severe invasive CAP.

#### Platelets in the Pathogenesis of Organ Dysfunction Associated With Chronic Bacterial Infection

In the case of chronic infection, Entezari et al. in an earlier study reported that levels of HMGB1 were significantly elevated in sputa and bronchoalveolar lavage fluid (BALF) from patients with cystic fibrosis (CF) chronically infected with *P. aeruginosa* (97). Although the cellular source of HMGB1 was not identified, it is nevertheless noteworthy that platelets appear to be intimately involved in the pathogenesis of CF (98, 99). In an extension of their clinical study, Entezari *et al*. developed a murine model of CF in which the gene encoding the CF transmembrane conductance regulator had been deleted (*CFTR* ^−/−^ mice). This murine model of CF enabled the authors to explore possible associations between elevated HMGB1 and impairment of pulmonary anti-bacterial host defenses in the airways of mice experimentally infected with *P. aeruginosa* via oropharyngeal aspiration (97). A monoclonal antibody (mAb) targeted against rat rHMGB1, or a dummy Ab, were administered to animals in the experimental and control groups, respectively. The authors observed that administration of the HMGB1-neutralizing antibody either prior to, or at the time of infection, protected both *CFTR* ^−/−^ and wild-type mice against influx of neutrophils into the airways and pulmonary damage in the setting of a decreased bacterial load (97). In addition, they also observed that: (i) BALF from CF patients suppressed the phagocytic activity of both murine peritoneal macrophages and a macrophage cell line (RAW264.7); and (ii) murine macrophages in which the *TLR4* gene had been inactivated were resistant to the anti-phagocytic action of HMGB1 (97).

#### Platelets in the Pathogenesis of Hyperacute Systemic Inflammation and Associated Organ Dysfunction in Severe, Invasive CAP

Evidence in support of this contention is derived in large part from studies that have investigated relationships between the numbers, dimensions and activation status of circulating platelets with prognosis in patients with severe, all-cause, invasive CAP.

With respect to alterations in the numbers of circulating platelets in patients hospitalized with severe CAP, both thrombocytopenia (100–106) and thrombocytosis (104–108), measured in most cases at the time of hospital admission, are associated with significantly increased mortality, either in-hospital and/or post-discharge. With respect to alterations in platelet turnover and reactivity, an increase in the mean platelet volume (MPV) measured as an increment over the first 4 days of hospital admission, is, according to one study, a significant predictor of mortality among adult patients admitted to an intensive care unit with severe pneumonia (109). In another recent study, the MPV/platelet count (MPV/PC) ratio, which interestingly is a marker of CVD, significantly predicted 30 day mortality in patients with pneumonia and acute ischemic stroke relative to those with acute ischemic stroke only (110).

With respect to systemic activation of platelets in severe pneumococcal CAP, Tunjungputri et al. described a strong association between expression of the platelet-binding pneumococcal adhesin, pblB, and 30 day mortality in hospitalized patients (*n* = 349) with proven (molecular/microbiological confirmation) pneumococcal bacteremia (111). *In vitro* experiments revealed a mechanistic interaction between expression of pblB on the pathogen and platelet activation. In this context, exposure of a pblB-expressing strain of the pneumococcus, but not a paired *pblB* gene-knockout strain, to isolated platelets resulted in increased expression of platelet CD62P, activation of GPIIb/IIIa, and formation of platelet/monocyte aggregates (111).

In the case of patients hospitalized with all-cause CAP, prolonged platelet activation detected at the time of hospital admission that persisted for up to 30 days has been described (112). In these studies, sustained platelet activation was measured according to prolonged upregulation of expression of CD62P and formation of platelet/leukocyte and platelet/monocyte heterotypic aggregates (112).

As mentioned above, approximately 30% of patients with severe CAP develop sepsis. In this context platelet gene expression (mRNA transcripts) has recently been measured in patients hospitalized with severe sepsis (*n* = 118, mean platelet counts 186 ± 92 vs. 287 ± 59 for 52 matched healthy subjects, *P* < 0.0001; MPV values comparable; sites of primary infection and causative pathogens not mentioned) (113). Within 24 h of onset of sepsis, both in humans and in mice, it was revealed that megakaryocytes initiate trafficking of mRNA encoding the *ITGA2B* gene (encodes integrin α-chain 2b) to platelets (113). Subsequent translation resulted in *de novo* synthesis of the αIIb component of integrin GPIIb/IIIa, as well as integrin activation, which were associated with mortality in both humans and mice (113).

Table 2 summarizes these various abnormalities of platelet numbers and reactivity in patients with severe CAP.

**Table 2:** Abnormalities of platelet numbers and reactivity in patients with severe CAP.

**Abnormality**	**Relationship to outcome**	**References**
Thrombocytopenia measured mostly at the time of hospital admission^*^	Significantly increased mortality either in-hospital or post-discharge	(100, 106)
Thrombocytosis also measured mostly at the time of hospital admission	Significantly increased mortality either in-hospital or post-discharge	(104–108)
Increasing mean platelet volume (MPV) measured over the first 4 days of hospital admission	A significant predictor of mortality in patients admitted to intensive care	(109)
Increased mean MPV/platelet count ration	A significant predictor of 30 day mortality in patients with ischemic stroke and pneumonia	(110)
Systemic activation of platelets with CAP caused by pblB-expressing strains of the pneumococcus	Increased 30 day mortality	(111)
Systemic activation of platelets that persisted throughout the entire course of hospital admission measured according to systemic levels of sCD62P, sCD40L, TxB2, and platelet heterotypic aggregates	Not mentioned in one study, associated with cardiovascular events in another	(112, 114)

* *With the exception of one study (111) all involve patients with all-cause CAP*.

### Platelets and the Pneumococcus

As alluded to above, the pneumococcus possesses several types of adhesin that promote binding of the pathogen to human platelets, which in the case of the FcGIIAR, TLR2, and pblB, are likely to trigger platelet activation and mobilization of antimicrobial peptides. Interestingly, however, it has recently been reported that the pneumococcus, unlike *E. coli* and *S. aureus*, is resistant to killing by TRAP-6 (thrombin mimetic)-activated human platelets or by exposure to platelet releasate (cell-free supernatant of TRAP-6-activated platelets); however, the anti-pneumococcal effects of platelet lysate were not reported (115). Although the mechanism of resistance of the pneumococcus to platelet-mediated killing was not established, it is noteworthy that platelets did not appear to survive exposure to the pathogen (115).

With respect to HMGB1/pneumococcus interactions, invasive pneumococcal disease is associated with elevated levels of this protein in sputum (116). Increased sputum levels of HMGB1, albeit of unknown cellular origin, are significantly associated with bacteremia, but not disease severity, indicative of a possible, albeit unproven, role for HMGB1 in promoting dissemination of the pneumococcus that possibly involves platelets (116).

Taken together with the various strategies utilized by the pneumococcus to evade NETosis (114, 117–120), a process triggered and amplified by platelet-dependent mechanisms, the aforementioned evidence suggests that the pneumococcus may be particularly adept at evading and even hijacking platelet-driven host defenses. In the setting of invasive pneumococcal CAP, the resultant misdirected, systemic pro-inflammatory/pro-thrombotic activities of platelets may contribute to the pathogenesis of the high prevalence of CV complications associated with this condition.

## Pathogenic Mechanisms of Cardiovascular Complications in Patients With CAP

Multiple mechanisms are likely to contribute to the pathogenesis of acute cardiac events in CAP, including pneumococcal CAP. These include the pro-thrombotic, pro-coagulant state associated with acute infection that encompasses platelet activation and production of pro-coagulants, NETosis and impaired anti-coagulant activity of the endothelium (14). Although it is clear that invasive CAP is associated with increased systemic reactivity of platelets, and with the occurrence of AMI, a mechanistic association between these events, while plausible, has nevertheless not been conclusively established. Given the therapeutic potential of adjunctive platelet-targeted therapies in severe CAP, this represents a topical and compelling theme of current clinical and laboratory infectious diseases research.

### Evidence From Clinical Studies Implicating the Involvement of Platelets in the Pathogenesis of Cardiovascular Events in Patients With All-Cause CAP

Cangemi et al. were among the first clinical researchers to identify a potential link between platelet activation and the development of CVEs in the setting of acute, albeit all-cause, CAP (114). The investigators studied 278 consecutive patients hospitalized for CAP, who were followed to discharge (114). They determined systemic levels of several platelet activation markers, including soluble P-selectin and CD40 ligand, thromboxane B2 (TxB2, a surrogate for TxA2) and high-sensitivity cardiac troponin T, together with measurement of electrocardiograms at 12 and 24 h intervals. Among 144 patients with elevated high-sensitivity troponin, 31 had signs of AMI and 113 did not, while baseline levels of the platelet markers were elevated in all patients with signs of AMI. Logistic regression analysis revealed that elevated soluble P-selectin (*p* < 0.001), soluble CD40 ligand (*p* < 0.001), TxB2 (*p* = 0.030), mean platelet volume (*p* = 0.037), the Pneumonia Severity Index (PSI; *p* = 0.030) and cardiac ejection fraction (*p* = 0.001) were independent predictors of MI (114). Interestingly, no significant differences in the occurrence of MI were detected between patients (*n* = 123) on aspirin prophylaxis [100 mg/day; inhibitor of cyclooxygenase 1 (COX-1)] and those that were not and aspirin-treated patients with MI had higher serum TxB2 levels than those without MI (*p* = 0.005).

The accompanying editorial raised several questions. Firstly, is platelet activation the cause of MI, given that aspirin therapy did not reduce the risk of MI, and recognizing that there are many pathways to platelet activation and simply interrupting one pathway may not be sufficient (120)? Secondly, is platelet activation simply a marker of disease severity, noting that the studies, including the current one, document that severe CAP is an independent risk factor for MI (120)? In addition, a vigorous debate ensued in the literature as to whether this study had in fact demonstrated a causal link between platelet activation and MI in patients with CAP. This contention was based on a number of issues, such as studying platelet activation rather than aggregation, the suitability of the platelet markers used, the possibility of underlying comorbidities being associated with elevated platelet reactivity, and the presence of renal failure, among others (121–123), to which the authors of the study and the editorial responded (124–127). Interestingly, one of the letters raised the issue of the use of statins as adjunctive therapy in severe CAP, a suggestion with which the authors of the initial study agreed and this is discussed more fully below (128).

A later study undertaken by the same authors involved consecutive patients with CAP admitted to a University Hospital in Rome who were followed up prospectively to discharge or death (129). The primary endpoint was death up to 30 days after admission, while the secondary endpoint was intra-hospital occurrence of non-fatal MI and ischemic stroke. One thousand and five patients (mean age 74.7 ± 15.1 years) were recruited of whom 390 were receiving aspirin (100 mg/day), while 615 were aspirin free. Overall, 16.2% of patients died [19 (4.9%]) among the aspirin users and 144 (23.4%; *p* < 0.001) among the non-users]. Non-fatal CVEs occurred in 4.9% of aspirin users and 8.3% of non-users (odds ratio 1.77; 95%CI 1.03–3.04; *p* = 0.040). Compared with patients taking aspirin the propensity score adjusted analysis confirmed that those not taking aspirin had a hazard ratio of 2.07 (95% CI 1.08–3.98; *p* = 0.029) for total mortality. An additional study by the same investigators was a *post hoc* analysis of patients presenting with septic shock in association with CAP and healthcare-associated pneumonia (130). The findings of this study suggested that use of both low-dose aspirin (100 mg/day) and macrolide antibiotics was associated with a lower mortality, possibly because of the anti-inflammatory effects of both the study drugs, together with the reduction in CVEs with aspirin (130).

This apparent link between excessive systemic activation of platelets activation and development of CVEs in CAP is supported by a more recent retrospective study of 351 hospitalized CAP patients, in which platelet counts were measured on admission and after 3–5 days (100). The authors noted that initial platelet counts were significantly lower in those patients who suffered MACE, which was defined as mortality, acute myocardial infarction or stroke, during the 1 year follow-up, compared with those that did not (225 × 10^9^/L vs. 261 × 10^9^/L; *p* = 0.036) (100). Those patients with a normal initial platelet count on hospital admission who subsequently developed thrombocytopenia (<150 × 10^9^/L) had a worse outcome, not only for all-cause mortality (HR 7.75; *p* = 0.002), but also for MACE (HR 7.4; *p* = 0.002) regardless of age, severity of pneumonia or comorbidity (100).

### Evidence Linking Platelet Activation to the Pathogenesis of Cardiovascular Events in Severe Pneumococcal CAP

Although the pneumococcus remains the most common and threatening bacterial cause of CAP, clinical studies linking platelet activation to outcome have invariably focused on mortality rates in all-cause CAP, rather than associations between platelets, pneumococcal infection and the prevalence of CVEs. Nevertheless, the study reported by Musher *et al*. has clearly identified that invasive pneumococcal disease carries a very high risk for development of serious CVEs (26), while that reported by Tunjungputri et al. has identified the association of severe pneumococcal infection with platelet activation and mortality (111).

Several experimental studies have also suggested a possible role for platelets in the pathogenesis of CVEs associated with severe pneumococcal disease. In this context, platelets, which capture, but do not kill, viable pneumococci, may act as vehicles that transport the pathogen to distant anatomical sites (47, 115).

Following translocation to the myocardium, the pneumococcus may intensify systemic platelet activation via interaction of these cells with various adhesins and other surface components of the pathogen, including complement activation products (40, 47–55).

In addition, release of pro-thrombotic/pro-inflammatory pneumolysin from disintegrating organisms also contributes to platelet activation. At sub-lytic concentrations, this pore-forming toxin induces Ca^2+^-dependent upregulation of expression of platelet CD62P, promoting homotypic platelet aggregation, heterotypic platelet/neutrophil aggregation and NET formation (131–133) that is exacerbated by mobilization of platelet HMGB1 (83, 84).

These events increase the risk of cardiac microvascular occlusion and myocardial dysfunction, a contention that is supported by findings that various biomarkers of platelet activation and neutrophil:platelet aggregates, as well as NETs and their constituents, such as histones, are major components of intravascular thrombi (134–136). In this context it is noteworthy that systemic histones, presumably derived from NETs, are associated with thrombocytopenia in critically ill patients, an association that has also been observed following experimental administration of histones to mice (137, 138). Although again focused on all-cause CAP and mortality (among other clinical end-points), the threat posed by intravascular NETosis in this condition has recently been underscored by the findings of a study communicated by Ebrahimi et al. (139). These authors observed that increased systemic levels of cell-free nucleosomes, a surrogate biomarker of NETosis, measured at the time of hospital admission, were associated with a 3.8-fold increased adjusted odds ratio of 30 day mortality (139).

Although the aforementioned mechanisms, together with the direct cytotoxic effects of pneumolysin on cardiomyocytes and resident cardiac macrophages (31–33), may underpin the pathogenesis of CVEs in acute, invasive pneumococcal infection, they do not explain the long-term cardiac sequelae in those who have seemingly recovered from this condition. Although unproven, persistent antigenemia has been proposed as a putative risk factor in this clinical setting, particularly in the case of the elderly, many of whom may have age-related platelet hyperreactivity and associated, persistent, low-grade systemic inflammation (38, 39).

Indeed, persistent antigenemia, particularly of pneumococcal capsular polysaccharides, for a duration up to 6 months post-infection, and possibly longer, has been detected in those who have recovered from an episode of pneumococcal CAP [reviewed in (13)]. Although the cellular reservoirs of pneumococcal antigen remain to be established, it is noteworthy that pneumolysin has been reported to interact with the mannose receptor C type 1 (MRC-1) expressed predominantly on M2-like anti-inflammatory macrophages and dendritic cells (140). Interaction of surface-expressed pneumolysin enables the pathogen to interact with MRC-1, resulting in uptake into non-lysozomal intracellular compartments that provide a safe haven for the pneumococcus (140).

**Figure 1:**
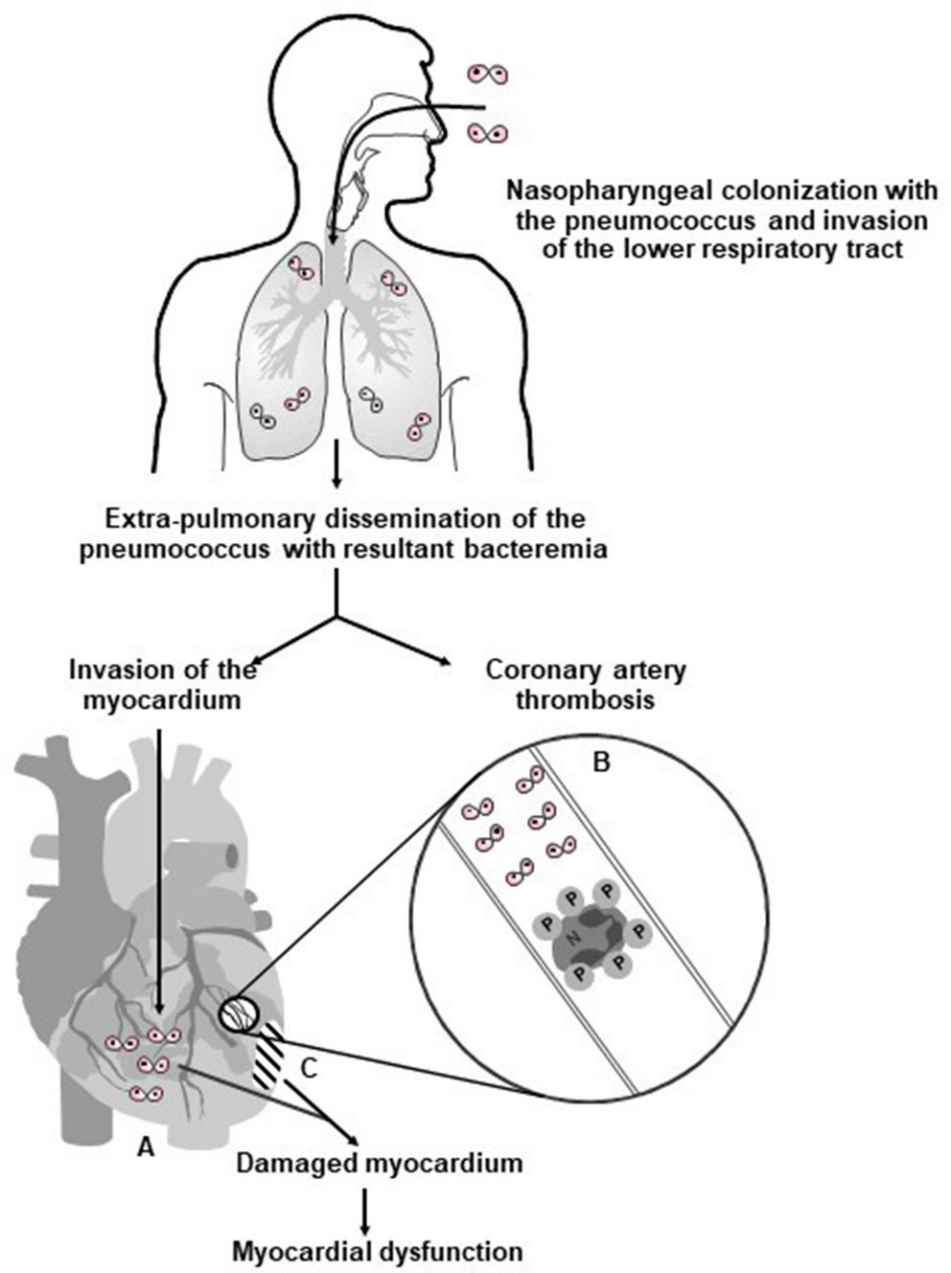
Proposed mechanisms involved in the pathogenesis of pneumolysin-mediated myocardial injury during invasive pneumococcal disease in humans. Following nasopharyngeal colonization, invasion of the lower respiratory tract, extra-pulmonary dissemination, and cardiac invasion by the pneumococcus (⊙⊙–symbol represents diplococci), intra-myocardial and intravascular release of pneumolysin (PLY) by the pathogen results in **(A)** PLY-mediated death and dysfunction of cardiomyocytes; **(B)** intravascular activation of platelets and neutrophils with resultant formation of pro-thrombotic/pro-NETotic neutrophil (N):platelet (P) aggregates (as illustrated in the magnification of an affected coronary arteriole/artery); and **(C)** development of myocardial damage and dysfunction. Reproduced with permission of Anderson et al. (28) under a Creative Commons License (https://creativecommons.org/licenses/by/4.0/).

Figure 1 summarizes the prominent role of pneumolysin in the pathogenesis of CVEs in pneumococcal CAP, encompassing platelet activation, as well as direct cardiotoxicity.

## Treatment of CAP

A detailed description of appropriate antibiotic, adjunctive, and supportive therapy in CAP is clearly beyond the brief of the current manuscript. However, it would seem appropriate to mention host- and pathogen-related risk factors that appear to predict the likelihood of a patient with CAP having a CVE, who could then be prioritized for personalized management with routine cardiac monitoring in the acute phase and targeted control of cardiovascular parameters regularly, both during and after hospital discharge (141, 142). This is followed by a section describing those aspects of antibiotic and adjunctive therapy that may relate to effects that these agents may have on platelet activity.

While CVEs have been documented to occur even in young, otherwise healthy, individuals, studies have indicated that older age, nursing home residence, pre-existing CVD, hyperlipidemia, hypoalbuminemia and higher pneumonia severity, such as assessed by the Pneumonia Severity Index (PSI), but also other markers of severity, appear to be important host risk factors (141, 143, 144). Viasus et al. (141) derived a simple rule based on demographic and clinical features that was helpful in identifying patients with CAP at higher risk of acute cardiac events. With regard to bacterial factors of importance, the latter study indicated that those patients with pneumococcal pneumonia appeared to be at greater risk of CVEs and we have highlighted previously in this manuscript additional studies that have noted the importance of pneumococcal bacteremia, and/or infection with pneumococcal serotypes that cause high-grade bacteremia as being most important in respect of this pathogen (30, 31). Other authors have suggested that patients with CAP due to *Staphylococcus aureus* and *Klebsiella pneumoniae* may also be at increased risk of CVEs (144). Lastly, investigators from Spain have noted that measurement of a range of cardiac biomarkers in the blood may be useful in identifying patients with CAP, at increased risk not only of early, but also long-term occurrence of CVE (12).

## Platelet-Targeted Adjunctive Strategies in CAP

Notwithstanding the potentially beneficial primary and secondary inhibitory effects of macrolide antibiotics on platelets, adjunctive strategies to antibiotics based on the use of recognized platelet-targeted therapies clearly have the potential, albeit relatively unexplored, to attenuate the development of both acute and delayed-onset, life-threatening CVEs in severe CAP. These include pharmacological strategies that directly target platelet activation and their mediators of thrombosis and inflammation, while direct targeting of platelet-activating bacterial toxins represents an additional, albeit indirect, approach. In the case of the former, potential options include the use of: (i) inhibitors of thromboxane synthesis such as aspirin and corticosteroids; (ii) antagonists of ADP-responsive P2Y12 purinergic receptors; and (iii) antagonists of thrombin-responsive proteinase-activated receptors 1 and 4 (PAR1/PAR4). Future, potential strategies include targeting of P2Y1 purinergic receptors, HMGB1 and necroptosis.

### Macrolide Antibiotics

Antibiotics are the mainstay of therapy in patients with CAP and must be administered as soon as the diagnosis is made to minimize the risk of mortality, while adjunctive therapies are used in those patients with more severe infection [reviewed in (145–147)]. In the case of severe infection, the antibiotic choice most commonly used is dual therapy, with the combination of a beta-lactam and a macrolide, usually azithromycin or clarithromycin (146). The rationale underpinning the use of macrolides in the combination therapy of all-cause CAP was initially based on the premise of expanded antibiotic coverage to include *Legionella pneumophila, Mycoplasma pneumoniae*, and *Chlamydia pneumoniae*. It is now well-recognized, however, that this latter class of antimicrobial agents possesses numerous additional activities that are beneficial in the treatment of CAP, including both antibiotic (148, 149) and non-antibiotic, neutrophil-targeted anti-inflammatory properties (148–151).

With respect to the former, macrolides as predominantly bacteriostatic agents that inhibit protein synthesis, appear to counteract the pro-inflammatory, platelet-activating activity of cell wall structures and toxins, such as pneumolysin, released following disintegration of bacterial pathogens by beta-lactam agents. This may be the mechanism by which most benefit is derived from combination therapy, where the macrolide is administered prior to the beta-lactam (152). In addition, macrolide antibiotics accumulate to extremely high levels in eukaryotic cells, including cells of the innate immune system and structural cells, countering both intracellular bacterial pathogens and development of antibiotic resistance (149).

With respect to non-antibiotic, anti-inflammatory effects of macrolides, we are aware of only one study that has investigated the effects of macrolides on platelet function. In this study reported by Tsoupras et al. *in vitro* exposure of washed rabbit platelets to therapeutically attainable concentrations of clarithromycin in particular, as well as azithromycin, resulted in significant attenuation of platelet-activating factor (PAF)-mediated aggregation of these cells, while the corresponding responses activated by thrombin were relatively unaffected (153). These findings await confirmation in the clinical setting.

The aforementioned beneficial effects of macrolides on reducing mortality in severe CAP imply that a similar effect may exist with respect to the occurrence of CVEs. To date, however, we are, unaware of stringently controlled clinical trials that have investigated the potential of macrolide-containing therapeutic regimens to ameliorate the development of CVEs in patients with severe CAP.

### Aspirin Therapy

One of the earliest studies undertaken to investigate the utility of aspirin in preventing the development of acute coronary syndrome (ACS) in patients with CAP was reported by Oz et al. (154). Using a multicenter, prospective, randomized trial design, these authors allocated patients who were hospitalized with CAP, and who had more than one risk factor for CVEs, to a control group (*n* = 94) or to a group that received treatment with aspirin (300 mg/daily, *n* = 91 patients) for 1 month. The primary endpoint was development of ACS within 1 month, while high-sensitivity cardiac troponin T (hs-cTnT) as a biomarker of cardiac dysfunction was measured on admission and after 48 h (154). The frequencies of development of ACS were 1.1% (*n* = 1) and 10.6% (*n* = 10) in the aspirin-treated and control groups, respectively (relative risk = 0.103; 95% CI = 0.005–0.746; *p* = 0.015). In addition, a significantly higher proportion of patients in the control group had elevated levels of hs-cTnT at 48 h post-hospital admission (55.3% vs. 35.2% for the control and aspirin-treated groups, respectively, *p* = 0.006). Concurrent diabetes mellitus and a Framingham cardiac disease risk score of >20 were significant risk factors for development of ACS. Although of interest, perceived imitations of the study included small numbers of patients, lack of pathogen identification and absence of data on circulating platelet counts and activation status (154).

As mentioned above, the study reported by Cangemi et al. indicated that patients with CAP who received low-dose aspirin prophylaxis prior to and during hospitalization experienced no significant in-hospital benefit with respect to either mortality or occurrence of non-fatal CVEs (114). However, in a follow-up study, to which a larger number of hospitalized patients with CAP was recruited, both 30 day mortality and in-hospital occurrence of non-fatal CVEs as primary and secondary endpoints, respectively, were significantly decreased in patients receiving low-dose aspirin prophylaxis (129). Two subsequent studies by the same authors, one in a small cohort of patients (*n* = 188) with CAP-associated septic shock (130) and a much larger multicenter study spanning four countries, these being Italy, USA, China and Japan, encompassing 1,295 patients with severe CAP, revealed that the combination of low-dose aspirin prophylaxis (≥100 mg/daily) and treatment with a macrolide antibiotic significantly improved the 30 day survival rate (155). In the latter study, the 30 day survival rate in patients treated with the aspirin/macrolide combination (*n* = 148) was significantly better (*p* = 0.002) than the corresponding rates observed in the subgroups of patients treated with aspirin without a macrolide (*n* = 237), macrolide without aspirin (*n* = 294) and aspirin/macrolide untreated (*n* = 616) (155).

The authors of the latter study, while acknowledging the limitation of its observational design, proposed that clinical benefit of the macrolide/aspirin combination in severe CAP results from a dual anti-inflammatory (macrolide) and anti-platelet (aspirin) mechanism of action (155).

On a cautionary note, however, a recently reported retrospective study from Denmark is noteworthy. The aim of this study, authored by Basille et al. was to determine the possible association between the use of non-steroidal anti-inflammatory drugs (NSAIDs) and clinical outcome in hospitalized patients with CAP (156). Of the 59,250 patients aged >15 years recruited to the study, which was conducted from 1997 to 2011, 27,714 had a history of usage of NSAIDs (current, new, long-term and former). The authors reported that users of NSAIDs hospitalized with CAP were at higher risk than non-users for development of pleuropulmonary complications, with younger patients and those with no comorbidities seemingly at highest risk (156). The authors conclude that this risk may result from masking of symptoms of serious disease and/or interference with host pulmonary defenses (156).

### Adjunctive Corticosteroid Therapy

Several recent meta-analyses have noted that adjunctive administration of corticosteroids (CS) to adult patients with severe CAP was associated with significant outcome benefits (157–160). In the latter study, there were differences documented in the mortality rates of the different corticosteroids, with prednisolone and methylprednisolone reducing total mortality, but use of hydrocortisone did not (160). With respect to the effects of CS on platelet function, older investigations noted differing results, which they attributed to different types and concentrations of CS used; however, in one study prednisolone was shown to have exquisite inhibitory properties on platelet function, which appeared to be related to selective regulation of ADP-activated P2Y12 receptor signaling, with the potential to attenuate vascular thrombotic episodes (161). In addition, Cangemi et al. who had previously reported an increased occurrence of AMI that was associated with elevated systemic levels of TxB2 in CAP patients (114), subsequently investigated the effects of CS on platelet activation *in vitro* and *in vivo*. In the first of two studies, these authors reported that betamethasone and methylprednisolone at concentrations of 150–600 nanograms/mL caused statistically significant inhibition ADP-activated aggregation of human platelets *in vitro*, in the setting of decreased activity of cytosolic phospholipase A_2_, and production of TxB2, seemingly consistent with an inhibitory effect on signaling mediated via activation of platelet P2Y12 and/or P2Y1 receptors (162). This was associated with levels of urinary 11-dehydro-TxB2 that were significantly higher (*p* < 0.001) in hospitalized CAP patients (*n* = 300) relative to those of control subjects (outpatients, *n* = 150) and independently predicted the occurrence of MI (162). These same investigators confirmed that CS use was associated with a lower incidence of MI in patients hospitalized with CAP (*n* = 758) in a retrospective record review of consecutively recruited patients followed prospectively to hospital discharge (163).

In the study alluded to above in which Ebrahimi et al. described an association between elevated biomarkers of intravascular NETosis and 30 day mortality in patients with severe CAP, it is noteworthy that these authors also noted that patients treated with prednisone demonstrated decreased NETosis and a beneficial outcome (139). However, others have recommended caution with regard to the routine use of CS other than perhaps for patients with severe CAP requiring ICU admission, citing the fact that many of the studies of CS use in CAP are relatively small, have some methodological issues, and the potential harmful effects need to be more fully elucidated; hence, while they concede that CS may have benefit in a small subset of cases with severe CAP, these cases have not yet been adequately identified (164). Other study results suggest that it is not only important to demonstrate benefits in stringently controlled randomized controlled trials, but also in the real world situation (165). It is hoped that current and ongoing studies of CS use in patients with CAP will clarify some of these issues (164, 166).

### P2Y12 Receptor Antagonists

Platelets possess two receptors for ADP, namely P2Y1 and P2Y12 receptors. Because ADP-mediated stimulation of platelets is dependent on co-activation of these receptors, blockade of only one receptor type may be adequate to attenuate responses initiated by exposure to ADP (167). Of the three pharmacological P2Y12 receptor antagonists currently in clinical use, namely clopidogrel, prasugrel and ticagrelor, which vary with respect to onset of action and efficacy (168), only clopidogrel and ticagrelor, to our knowledge, have been evaluated in the setting of CAP

In the first of these, Gross et al. in a retrospective study spanning the period 2001–2005, investigated firstly the frequency of development of CAP in subjects who had a history of having been prescribed clopidogrel on at least six occasions (*n* = 5,166) in comparison with those who had not (*n* = 52,809) (169). Secondly, assessment of the requirement for hospital admission following development of CAP in the clopidogrel-treated and untreated groups, as well as indices of disease severity, including mortality, intensive care unit (ICU) admission, need for mechanical ventilation, development of sepsis, acute respiratory distress syndrome/acute lung injury, but not, somewhat surprisingly, development of CVEs (169). Surprisingly, the authors reported that both the incidence of CAP, as well as the frequency of hospital admission, were significantly higher in the clopidogrel-treated group, with trends, albeit not attaining statistical significance, indicative of less severe disease (169).

The Platelet inhibition and patient Outcomes (PLATO) study was a multicenter, double-blind, randomized trial designed to compare the effects of ticagrelor (180 mg loading dose, 90 mg twice daily thereafter) and clopidogrel (300–600 mg loading dose, 75 mg daily thereafter) on the prevention of CVEs in patients admitted to hospital with an ACS (170). The study recruited a total of 18,624 patients allocated in equal numbers to receive treatment with either ticagrelor or clopidogrel for 1 year. The findings of the study revealed that treatment with ticagrelor, as opposed to clopidogrel, significantly decreased the rate of death from MI or stroke (170). A subsequent secondary analysis of the data emanating from this trial was focused on the prevalence of pulmonary adverse events, mostly pneumonia, as well as associated mortality, in both treatment arms (171). This analysis revealed significantly lower prevalence rates of pulmonary adverse events and sepsis, as well as associated mortality, in the ticagrelor-treated group. The authors concluded that the mortality risk following pulmonary adverse events and sepsis in ACS patients appears to be lower in those receiving ticagrelor (171). An important caveat, however, is that all patients in both treatment groups also received aspirin (175 mg/daily), indicating that the combination of ticagrelor and aspirin may in fact be most effective, as opposed to ticagrelor alone.

In a more recent study known as the XANTHIPPE study (Examining the effect of Ticagrelor on Platelet Activation, Platelet-Leukocyte Aggregates and Acute Lung Injury in Pneumonia), Sexton et al. investigated the effects of ticagrelor on systemic biomarkers of platelet activation, as well as pulmonary function in hospitalized patients with pneumonia [CAP and hospital-acquired pneumonia (HAP)] (172). The study design was a randomized, placebo-controlled trial with 30 patients in each of the placebo-treated (21 CAP and 9 HAP) and ticagrelor-treated (17 CAP and 13 HAP) groups. Patients who were recruited within 48 h of diagnosis, were treated for 7 days, or until discharge (172). Biomarker and lung function measurements were performed at baseline and after 1, 2, and 7 days of treatment or at the time of discharge and, finally, after 30 days. Briefly, administration of ticagrelor was associated with significant decreases in systemic levels of IL-6, as well as in ADP-activated formation of platelet-leukocyte aggregates *ex vivo* that was evident from day 1. In addition, trends were evident toward improved pulmonary function and need for supplemental oxygen (172). Similar effects of ticagrelor were observed in a murine model of experimental sepsis, including significantly decreased mortality (172). Limitations of this study identified by the authors include the small numbers of patients, all with moderate disease (172).

### Dual Anti-platelet Therapy

To our knowledge there are no stringently controlled studies in the current literature that have described the effects of dual anti-platelet therapy, usually aspirin in combination with a P2Y12 receptor antagonist, in the clinical setting of severe CAP. This may relate to concerns about the risk of bleeding complications.

### Proteinase-Activated Receptor 1 (PAR-1) Antagonists

Vorapaxor remains the only selective antagonist of thrombin-activated PAR-1 receptors. To our knowledge, however, this agent has not been used in the treatment of severe CAP, either alone or in combination with aspirin and/or a P2Y12 receptor antagonist.

### Statins

These cholesterol-lowering agents, which target the enzyme 3-hydroxy-3-methyl-glutaryl CoA reductase, suppress platelet reactivity by several mechanisms that have been reviewed by us elsewhere (173). These include: (i) inhibition of the formation of oxLDL-C, which triggers platelet activation via interaction with the scavenger receptors, LOX-1 and CD36, both of which are expressed on platelets (174, 175); (ii) inhibition of PLA_2_, resulting in decreased release of TxA2 (176); (iii) decreased expression of the adhesion molecule CD40L (177); and (iv) although largely unexplored, by lowering membrane cholesterol concentrations, statins may attenuate the pro-inflammatory/pro-thrombotic activities of cholesterol-binding toxins such as pneumolysin. In this context administration of simvastatin at a dose of 20 mg/daily for 6 weeks to hypercholesterolemic patients resulted in significantly decreased concentrations of cholesterol in the plasma membranes of isolated blood neutrophils (178). These anti-platelet activities of statins have also been described in the clinical setting, albeit in newly-diagnosed patients with primary hypercholesterolemia treated with simvastatin (179). In this study, Barale et al. observed that aside from improved lipid profiles, administration of simvastatin was associated with significant reduction in ADP- and collagen-activated platelet aggregation *ex vivo*, as well as reductions in systemic biomarkers of platelet activation, including sCD62P, sCD40L, platelet-derived growth factor BB and RANTES. With respect to clinical studies in patients hospitalized with CAP, these have been mostly of a retrospective design in patient cohorts that had received statins prior to hospital admission, the broad consensus finding being that of a modest-to-moderate beneficial effect on mortality (173).

More recently, Sapey et al. *r*eported the findings of a pilot, randomized, controlled clinical trial designed primarily to investigate the effects of short-term administration of simvastatin on neutrophil function and clinical outcomes in elderly patients (*n* = 62, aged > 55 years) admitted to a secondary care facility for “milder” CAP (180). Patients received either simvastatin (*n* = 32 patients, 80 mg daily for 7 days or until discharge) or a placebo (*n* = 30 patients), together with a macrolide antibiotic. Neutrophil functions, measured using isolated peripheral blood neutrophils, were performed on treatment day 4 and included, most importantly, measurement of chemoattractant-stimulated NETosis, as well as IL-8-induced chemotaxis, apoptosis and systemic neutrophil elastase activity, while clinical assessment was based on changes in the sequential organ failure assessment (SOFA) score. Treatment with simvastatin was associated with statistically significant decreases in NETosis (*p* = 0.0034) and systemic elastase activity (*p* = 0,001), while chemotactic responsiveness increased significantly and apoptosis was unaffected (180). Although, as expected, SOFA scores were relatively low in this cohort of CAP patients, they nevertheless decreased significantly (*p* < 0.026) in simvastatin-treated patients. With respect to long-term benefit, analysis of readmission and survival data as a composite endpoint revealed significant increases in hospital-free survival at both 180 and 365 days (*p* = 0.03 for both).

The authors, while conceding that the findings of this small proof-of-concept study require confirmation in large, stringently controlled multicenter trials, contend that elderly persons with mild CAP may be the principal beneficiaries of statin-based adjuvant therapy. They propose that statins may induce the transition of systemic neutrophils from a harmful, possibly platelet-driven, phenotype to a protective anti-inflammatory phenotype (180).

### Liposomes That Target CAP Pathogen-Derived, Cholesterol-Binding, Pore-Forming Toxins

Liposomes that contain a 1:1 mixture of cholesterol:sphingomyelin have been developed as a therapeutic strategy to counter the damaging activities of bacterial cholesterol-binding, pore-forming toxins such as pneumolysin (181). When administered to mice with severe, experimental invasive pneumococcal disease, these liposome preparations significantly attenuated the development of myocardial injury (34). Following from these promising earlier studies, a Swiss Biotechnology company, COMBIOXIN SA, developed a therapeutic liposome preparation known as CAL02 (182). As described by the developers “CAL02 consists of a mixture of liposomes that create artificially large and stable liquid-ordered lipid microdomains that function as docking sites for a large range of bacterial toxins” (181, 182).

CAL02 has recently been evaluated in a double-blind, placebo-controlled, randomized phase I clinical trial, encompassing six intensive care units in France and Belgium and a total of 19 patients with severe pneumococcal pneumonia 14 and 5 of whom received CAL02 or placebo, respectively (182). Although primarily focused on safety and tolerability issues, both of which were “promising”, it is noteworthy that the mean SOFA scores at 8 days decreased by 69.0 and 29.2% in the CAL02- and placebo-treated groups, respectively, while the corresponding figures for the occurrence of atrial fibrillation were 14.3% (2/14) and 40% (2/5) (182). These findings provide a basis for further clinical evaluation of CAL02 in larger clinical trials.

### Future Platelet-Targeted Strategies

These include pharmacological and biological agents that target ADP-activated P2Y1 receptors on platelets as well as platelet-derived, pro-inflammatory HMGB1. In addition, inhibitors of necroptosis may indirectly attenuate the harmful effects of platelet activation in the setting of severe pneumococcal disease.

#### P2Y1 Receptors

Although P2Y12 and P2Y1 receptors appear to harmonize with respect to ADP-mediated platelet activation, Amison et al. have recently described a novel mechanism involving only P2Y1 receptors that promotes platelet-dependent leukocyte recruitment at sites of inflammation (183). A P2Y1 receptor-mediated signaling pathway that is distinct from that involving phospholipase C and initiation of platelet aggregation drives this novel mechanism of platelet-dependent neutrophil chemotaxis. Although selective pharmacological inhibitors of P2Y1 receptors are available for use in experimental settings, none is yet available for clinical application.

#### HMGB1

Strategies targeting this pro-inflammatory protein have been the subject of a very recent review (184). Amongst others, these include pharmacological agents such as metformin that inhibit the translocation of HMGB1 from the nucleus to the cytosol that is likely to be most relevant to megakaryocytes, as well as direct targeting of the protein by monoclonal antibodies, such as m2G7 that target the interaction of HMGB1 with TLR4 and RAGE (184).

#### Inhibitors of Necroptosis

Very recently, Beno et al. using high-resolution echocardiography, observed that administration of ponatinib, a novel multi-tyrosine kinase inhibitor that attenuates necroptosis, to mice with experimentally induced severe pneumococcal disease, resulted in significant attenuation of long-term cardiac damage (185, 186). Although these cardio-protective effects of ponatinib were attributed to attenuation of pneumolysin-mediated cardiomyocyte necroptosis, it is also noteworthy that toxin-induced platelet activation and resultant microvascular occlusion represents an additional cause of cardiac necroptosis.

These various anti-platelet strategies are summarized in Table 3.

**Table 3:** Potential pharmacological and biological strategies to counter platelet activation in severe community-acquired pneumonia.

**Agent**	**Mechanism of action**	**References**
Macrolide antibiotics	• Counter the pro-inflammatory activities of beta-lactam antibiotics • May directly suppress platelet activation • May suppress platelet-driven pro-inflammatory activity of neutrophils	(149–153)
Aspirin	Inhibits TxA2-mediated autocrine activation of platelets	(129, 130, 154, 155)
Corticosteroids	Suppress synthesis of TxA2 via inhibition of cytosolic phospholipase A2	(161–163)
P2Y12 receptor antagonists (ticagrelor)	Attenuate ADP-mediated platelet activation	(171, 172)
Dual anti-platelet therapy (aspirin + P2Y12 receptor antagonists)	Untested in the clinical setting	–
Proteinase-activated receptor 1 (PAR-1) antagonists (vorapaxar)	Untested in the clinical setting	–
Statins	• Inhibit the formation of platelet-activating oxLDL-C • Inhibits phospholipase A2 and formation of TxA2 • Decreased expression of CD410L on platelets	(173–177, 179, 180)
Cholesterol-containing liposomes	Neutralize pneumolysin-mediated platelet activation	(34, 181, 182)
P2Y1 receptor antagonists	Not yet available	(183)
HMGB1 antagonists (metformin; monoclonal antibody, m2G7)	Not yet evaluated in CAP	(184)
Inhibitors of necroptosis (ponatinib)	Attenuate cardiotoxicity in a murine model of invasive CAP	(185)

## Conclusions

It is quite clear from this review, that platelets play an important role not only in host defense against infections, including, but not limited to CAP, but that platelet activation may also contribute to some of the potentially life-threatening complications that occur in patients with CAP, such as the acute CVEs. The involvement of platelets in the pathogenesis of these acute CVEs, exacerbated by bacterial invasion of the myocardium and release of cell wall components and toxins, such as pneumolysin, in the case of the pneumococcus, is relatively well-established. However, the events that are associated with the pathogenesis of the long-term cardiac events are less clear. Nevertheless, evidence is emerging that persistent antigenemia predisposes to a persistent, systemic pro-inflammatory/prothrombotic phenotype that is associated with an ongoing risk of future CVE. Although various, mainly pharmacological, platelet-targeted adjunctive therapies have been identified, evaluation of these in the clinical setting has been somewhat fragmented, necessitating future stringent assessment in well-controlled, definitive clinical trials.

## Author Contributions

Both authors contributed equally to the conceptualization and compilation of the manuscript and approved its submission.

## Conflict of Interest

The authors declare that the research was conducted in the absence of any commercial or financial relationships that could be construed as a potential conflict of interest.
